# The fecal microbiota of the mouse-eared bat (*Myotis velifer*) with new records of microbial taxa for bats

**DOI:** 10.1371/journal.pone.0314847

**Published:** 2024-12-05

**Authors:** Hanya D. Arellano-Hernández, Leslie M. Montes-Carreto, José Antonio Guerrero, Esperanza Martinez-Romero

**Affiliations:** 1 Labotarorio de Monitoreo y Conservación de Fauna, Facultad de Ciencias Biológicas, Universidad Autónoma del Estado de Morelos, Cuernavaca, Morelos, México; 2 Laboratorio de Ecología Genómica, Centro de Ciencias Genómicas, Universidad Nacional Autónoma de México, Cuernavaca, Morelos, México; Institut Pasteur de Madagascar, MADAGASCAR

## Abstract

Studies on the fecal microbiome of wild animals reveal valuable information on the feeding habits of the host and the possible roles of bacteria in digestion. In this work we characterized the fecal microbiota of seven male and seven female *Myotis velifer* bats using the V3-V4 regions of the 16S rRNA gene. Fecal samples were collected at the El Salitre cave in Mexico. We obtained 81 amplicon sequence variants, identifying four phyla, 12 families and 14 genera for females and seven phyla, 21 families and 26 genera for males. The phylum Synergistota is reported for the first time in bats. The most abundant phyla were Pseudomonadota and Fusobacteriota. Male feces showed a greater taxonomic richness than those from females. This study revealed that the fecal microbiota of *M*. *velifer* had a unique and more diverse composition compared to the microbiota reported for other bats. We identified 24 families and two abundant genera *Cetobacterium* and *Haematospirillum* in both males and females. *Cetobacterium* may produce vitamin B12 that is not produced by animals and *Haematospirillum*, which has been reported as an emerging human pathogen, may produce non-volatile organic acids. These genera had not been previously reported in the bat microbiota.

## Introduction

Typically, mammals establish symbiotic relationships with microbes that inhabit their skin, mucous membranes, and gastrointestinal tract, among others [1]. The microbiota is defined as a community of microorganisms residing within a host organ or tissue. The intestinal microbiota is species-rich and may have diverse capabilities to produce vitamins, aminoacids, short-chain fatty acids (SCFAs), enzymes, and neuroactive molecules such as serotonin [2–4]. It will, therefore, have effects on the host’s health [5], physiology [2, 6], sex and reproductive success [7, 8] and lifestyle [6, 9]. Bacterial communities are influenced by the same factors leading to highly dynamic interactions [10]. The gut microbiome encodes enzymes such as hydrolases necessary for the degradation of ingested food [3, 11–13]. For example, gut bacteria in insectivorous bats provide chitinases to break down chitin, the main component of insect exoskeletons [13]. In hematophagous bats, proteases facilitate protein digestion, absorption and metabolism [14]. Studies of wildlife microbiota have recently gained popularity due to their importance in host health, evolution, and ecology [2, 11, 15]. Microbial ecology has been revolutionized by the development of culture-independent techniques using shotgun sequencing [16] for metagenomics or mass sequencing of rRNA amplicons [17, 18]. These advancements enable researchers to assess the composition, diversity, structure, and functionality of microorganisms associated with their hosts [5, 11, 19]. One way to explore the intestinal microbiota noninvasively is by studying the fecal microbiota [20]. Though it has been argued that such samples are not representative of the gastrointestinal tract [21], fecal sampling has proven to be the most convenient non-invasive method, as it allows for repeated sampling of individuals over time, ensuring rapid collection and intact preservation [20, 22].

Bats, as the second most diverse and ecologically relevant group of mammals after rodents [23], harbor a variety of microorganisms that can be either beneficial or pathogenic [24–27]. In Mexico, 142 bat species have been identified, 100 of which are insectivorous [28–30]. Studies show that bats play important roles in ecosystems such as plant pollination, seed dispersal [31, 32] and control of insect populations by consuming large numbers of nocturnal insects, even consuming up to 150% of their body weight in one night [33]. Therefore, by consuming large quantities of insects, including crop pests and vectors of various diseases of medical importance [34, 35], bats provide economic, social and health benefits. They also act as natural fertilizers due to the nutrient richness of their guano (feces), which contains nitrogen, phosphorus, and microorganisms that can act as bioremediators, nematicides, and fungicides [36]. In this way, guano can be used as a fertilizer in crop fields or horticulture, potentially leading to economic savings by reducing the use of chemical pesticides and promoting sustainable agricultural practices [34].

Compared to the microbiota of non-flying mammals, especially herbivores [11, 37], bat microbiota exhibits less taxonomic diversity [38, 39]. The bat microbiota is dominated by the metabolically diverse bacterial phyla Pseudomonadota, Bacillota and Bacteroidota [24, 26, 38, 40]. At lower taxonomic levels, the composition of the gut microbiota of bats seems to be determined by the diet with specific bacteria linked to dietary specialization such as insectivory, frugivory, nectarivory, carnivory, or sanguivory diets [22, 24, 38, 41–44], suggesting a potential role for bacteria in nutritional and ecological differentiation [38]. When comparing different bat species, phytophagous (frugivorous and nectarivorous) species tend to harbor genera such as *Weissella*, *Ureaplasma*, *Klebsiella*, *Enterobacter*, *Escherichia*, *Enterococcus*, and *Fructobacillus* [24, 45], whereas insectivorous species are characterized by genera such as *Plesiomonas*, *Enterococcus*, *Lactobacillus*, *Bacillus*, *Lactococcus*, *Paeniclostridium*, *Undibacterium*, *Serratia* and *Yersinia* [21, 24, 26, 42, 46, 47]. In addition, differences in microbial composition between the sexes have been observed in nectarivorous [8] and frugivorous [48] species.

The mouse-eared bat (*Myotis velifer*) has a wide distribution on the American continent (from northern Kansas to Honduras and Guatemala). It is classified as a species of least concern according to red list of the IUCN [49]. It plays an important ecological role as a pest controller, consuming large quantities of nocturnal insects such as Coleoptera, Homoptera, Diptera, Lepidoptera, Hemiptera, among others [50–52]. The goal of this study was to describe the fecal microbiota of *Myotis velifer*, evaluate its richness and abundance, and compare the microbial diversity between females and males and between sexually active and inactive males.

## Materials and methods

### Study site

The collection of the samples was at “El Salitre” cave, located in the municipality of Tlaltizapan in the state of Morelos, Mexico (18° 45’00” N and 99°11’24” W) at an altitude of 1,100 meters above sea level. It is surrounded by patches of deciduous forest alternating with secondary vegetation, croplands, and grasslands. The cave has an entrance ~1.8 m high and ~3.5 m wide and consists of three chambers with a total length of 225 m. The temperature inside the cave is 20–25°C and humidity is 79–99% [53].

### Collection of individuals and feces

Access to the cave and the capture of bats was authorized by the Dirección General de Áreas Naturales Protegidas of Morelos, Mexico. We captured 14 *Myotis velifer* bats (seven males and seven females) with a 6 m long mist net placed at the cave entrance, between 10:00 p.m. and 11:30 p.m. on August 27, 2022, during the rainy season of the year. Each of the captured bats was placed individually in a clean, non-sterile blanket bag to ensure air circulation and prevent suffocation. The collection of feces began at 2:00 a.m., after having kept the bats in the individual bags for around two and a half hours. To avoid possible contamination between fecal, nitrile gloves were sanitized with 70% alcohol after handling each bat sample. Feces from each individual were stored in different sterile 1.5 ml Eppendorf tubes with DNA/RNA shield to preserve the DNA integrity at room temperature until arrival at the laboratory, where they were stored at -20°C until processing. In addition, standard morphological measurements of bats were taken for their taxonomic identification according to the Guide for the Identification of Mexican bats [54]: forearm length (electronic vernier in mm), body mass (with a 100 g spring balance), age category (juvenile or adult) estimated according to ossification of the wing bones (metacarpals and phalanges) and the condition in which they were found (males: scrotum, inguinal or abdominal testicles, females: lactating, pregnant or inactive). Finally, they were released at the entrance of the cave. We followed the protocols of the Animal Care and Use Committee of the American Society for Mammalogists [55] while handling bats.

### DNA extraction from feces

DNA was extracted from 14 fecal samples using the Wizard Genomic DNA Purification kit (Promega, USA) following the manufacturer’s instructions. The negative control was nuclease-free water. From each of the stool samples collected in sterile 1.5 ml tubes, ~180 mg was placed in a new tube and macerated using a sterile pestle. Subsequently, the extracts were purified with the High Pure PCR Template Preparation Kit (Roche, Germany). DNA concentration was quantified using a NanoDrop spectrophotometer at wavelengths of 230, 260, and 280 nm and DNA was observed in 1.2% agarose gels.

### Sequencing

The hypervariable regions (V3-V4) of the 16S rRNA molecular marker were amplified by PCR for each sample using specific primers 518 (5´-CCAGCAGCCGCGGTAATACG3´) F and 800 (5´TACCAGGGTATCTAATCC-3´) R [56]. The PCR reactions (50μL) contained 2μL of DNA, 25μL of Dream Taq PCR Master Mix (2X) (Thermo Scientific, USA), 20.5 μL of nuclease-free water, 0.25μL of bovine serum albumin (BSA), 1.25μL of dimethylsulfoxide (DMSO) and 0.5μL of each primer. Samples were amplified using a denaturation protocol at 94°C (5 min), followed by 30 cycles of 94°C (60 s), 53°C (60 s), and 72°C (60 s) and a final extension (72°C, 10 min). Finally, the amplified products were sequenced at Macrogen with Illumina NovaSeq 6000 paired-end 2x300 bp with reported adapters [56].

### Bioinformatics analysis

The raw reads were processed using the FastQC v0.12.0 program to evaluate their quality. Once this process was completed, the Fastp v0.23.4 program [57] was used to clean the sequences, trim adapters, and filter by quality with a Quality Score ≥ Q28. Using the QIIME2-2023.9 tool [58], amplicon sequence variants (ASVs) were obtained using DADA2 [59]. Taxonomic assignment of ASVs was obtained using feature-classifier [60], classify-sklearn [40, 58] using full-length sequences from Greengenes2-2022.10 [18] as reference sequences. Samples with unclassified bacteria were not considered in the further analysis.

The relative abundance of microbial taxa was calculated by dividing the total number of sequences of each phylum, family, and genus, by the total number of sequences of all groups for each taxonomic level, to recognize the most abundant taxa from the of *M*. *velifer* microbiota [61].

### Analysis of alpha and beta diversity

To ensure that the richness and diversity of the samples are representative of the fecal microbiota, we performed a coverage analysis of the different groups compared [11, 62].

Microbial richness and taxonomic diversity between males and females, and sexually active and inactive males, at the genus level were estimated using Hill numbers q (qD) [63], where q = 0 corresponds to species richness, q = 1 corresponds to the exponent of Shannon entropy (effective number of common elements), and q = 2 corresponds to the inverse of Simpson’s index (effective number of dominant elements [64, 65]. The qD diversity values, sample coverage, and their confidence intervals were obtained with the iNEXT package in R [66] using as criteria the maximum number of contigs of the samples (31,261 sequences) as the endpoint and a 95% confidence interval (CI) with 1,000 bootstraps to construct rarefaction curves. Variation in overall composition for phylogenetic diversity, beta- weighted diversity metric UniFrac [67] as well as principal coordinate analysis (PCoA) were estimated using diversity in QIIME2-2023.5 [58]. For taxonomic beta diversity, a similarity analysis (ANOSIM) was performed between groups (*M*. *velifer* males and females and sexually active and inactive males) based on the Bray-Curtis similarity metric and with 1000 permutations, using Past software v217b [68].

## Results

The total number of bats collected was 14, of which seven were females and seven were males. The reproductive condition in which they were categorized was as follows: seven inactive females and four inactive males, and three active males with scrotal testicles. All individuals were adults.

A total of 1,162,492 raw reads were obtained from the 14 samples. After cleaning, 867,768 reads remained, preserving an average of 95% of the sequences per sample. After taxonomic assignment, 84 amplicon sequence variants (ASVs) with a length range of 423 bp were identified.

### *Myotis velifer* fecal microbiota

**Fig 1:**
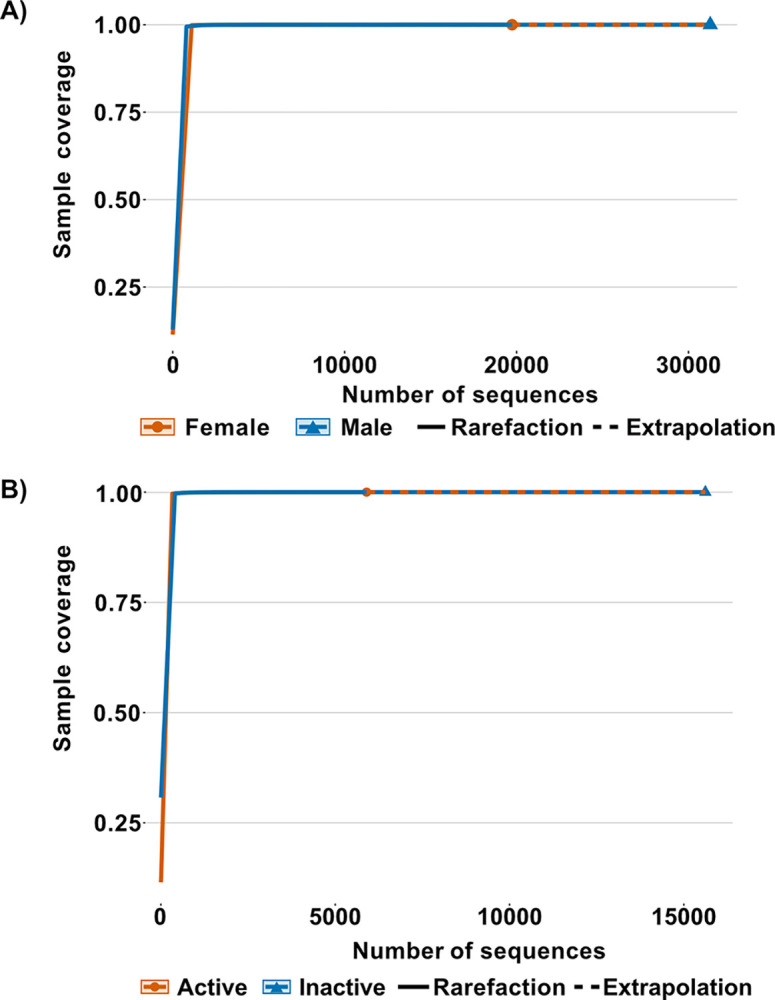
Sample coverage (SC) and number of sequences. (A) Rarefaction curves based on sample coverage by sex. (B) Rarefaction curves based on sample coverage by active and inactive males.

We obtained a total of seven phyla, 24 families (S1 Fig) and 31 genera in the fecal microbiota of the vespertilionid *M*. *velifer*. In both male and female samples, bacterial genera showed 100% sample coverage for q = 0, q = 1 and q = 2 demonstrating that diversity is complete for each sample (Fig 1A and 1B). Then, the comparisons of the diversity were carried out directly according to their confidence intervals [62].

**Fig 2:**
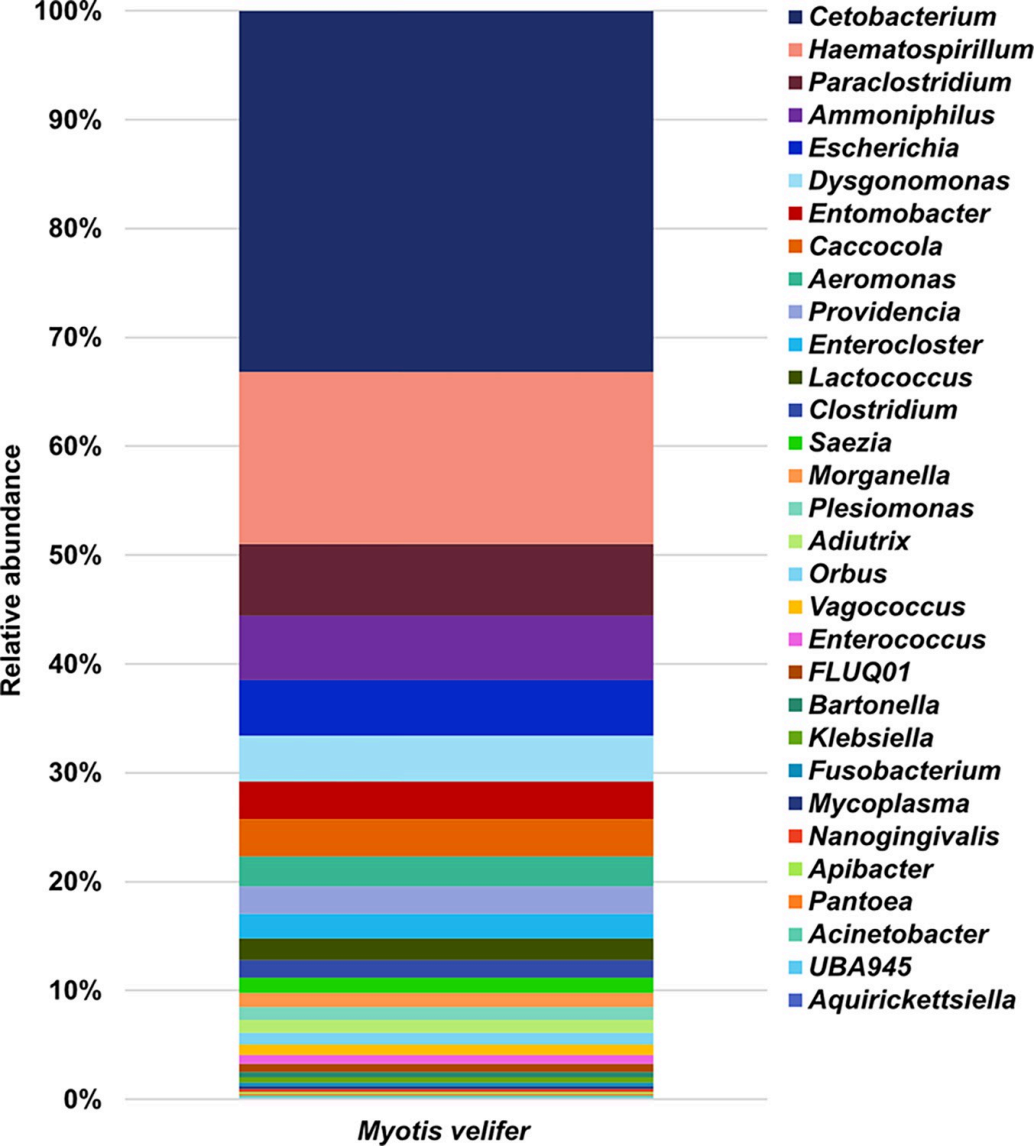
Bacterial genera found in the fecal microbiota of *Myotis velifer*. Percentage of relative abundance of the genera from domain Bacteria in the fecal microbiota of *M*. *velifer*. The genera are organized by relative abundance, with the most abundant at the top and the least abundant at the bottom.

Some of the most dominant genera were *Cetobacterium* (33%), *Haematospirillum* (16%), *Paraclostridium* (7%), *Ammoniphilus* (6%), *Escherichia* (5%), *Dysgonomonas* (4%), *Entomobacter* (3%), *Caccocola* (3%), *Aeromonas* (3%), *Providencia* (3%), *Enterocloster* (2%), *Lactococcus* (2%), *Clostridium* (2%), *Saezia* (1%), *Morganella* (1%), *Plesiomonas* (1%), *Orbus* (1%), *Adiutrix* (1%) and *Vagococcus* (1%) (Fig 2 and S2 Fig).

### Fecal microbiota by sex

**Fig 3:**
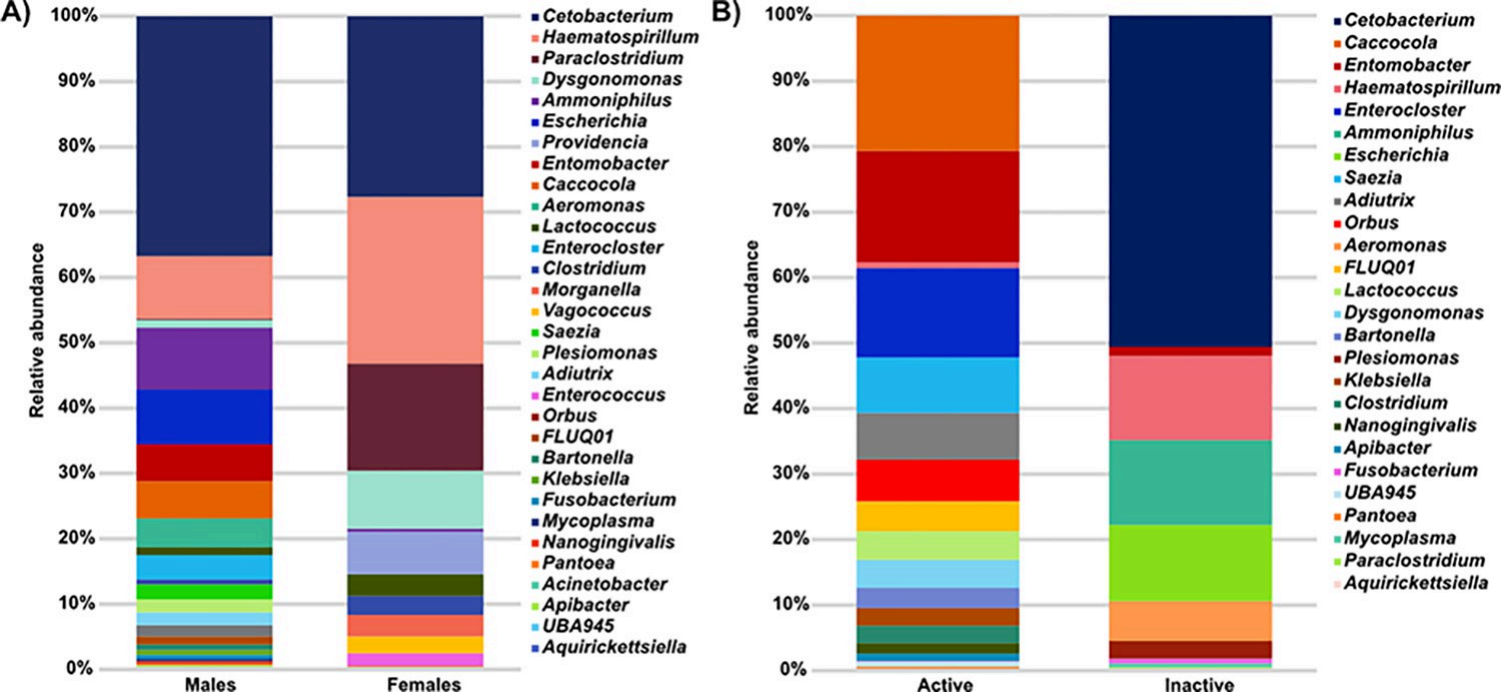
Bacterial genera from the fecal microbiota of *M*. *velifer* per sex and sexually active and inactive males. (A) Relative abundances of the bacterial genera present in the fecal microbiota from *M*. *velifer* males and females. (B) Relative abundances of bacterial genera present in the fecal microbiota of reproductively active and inactive *M*. *velifer* males. The genera are organized by relative abundance, with the most abundant at the top and the least abundant at the bottom.

In the fecal microbiota from females, 14 genera were found, the dominant ones were *Cetobacterium* (28%), *Haematospirillum* (25%), *Paraclostridium* (16%) and *Dysgonomonas* (9%) (Fig 3A). In males, 26 genera were found, nine of which are shared with the females. Among the most abundant were *Cetobacterium* (37%), *Haematospirillum* (10%), *Ammoniphilus* (9%) and *Escherichia* (8%) (Fig 3A).

In the fecal microbiota of the reproductively active males, a total of seven phyla were found, with Pseudomonadota (39%), Bacillota (21%) and Synergistota (21%) being dominant. We identified 17 families, and the most abundant ones were Synergistaceae (21%), Acetobacteraceae (17%) and Lachnospiraceae (14%) as well as 19 genera, *Caccocola* (21%), *Entomobacter* (17%), *Enterocloster* (14%) and *Saezia* (8%) as the most abundant (Fig 3B). As for sexually inactive males, a total of three phyla were found, with Fusobacteriota (51%), Pseudomonadota (35%), Bacillota (14%) as the dominant ones. Nine families were identified, among the most abundant were Fusobacteriaceae (51%), Enterobacteriaceae (14%) and Paenibacillaceae (13%); a richness of 11 bacterial genera was observed, of which four are shared with the active ones. The most abundant genera for these individuals were *Cetobacterium*, *Ammoniphilus*, *Haematospirillum* and *Escherichia* (51%, 13%, 13%, 12%, respectively) (Fig 3B and S3 Fig).

### Alpha diversity analysis

**Fig 4:**
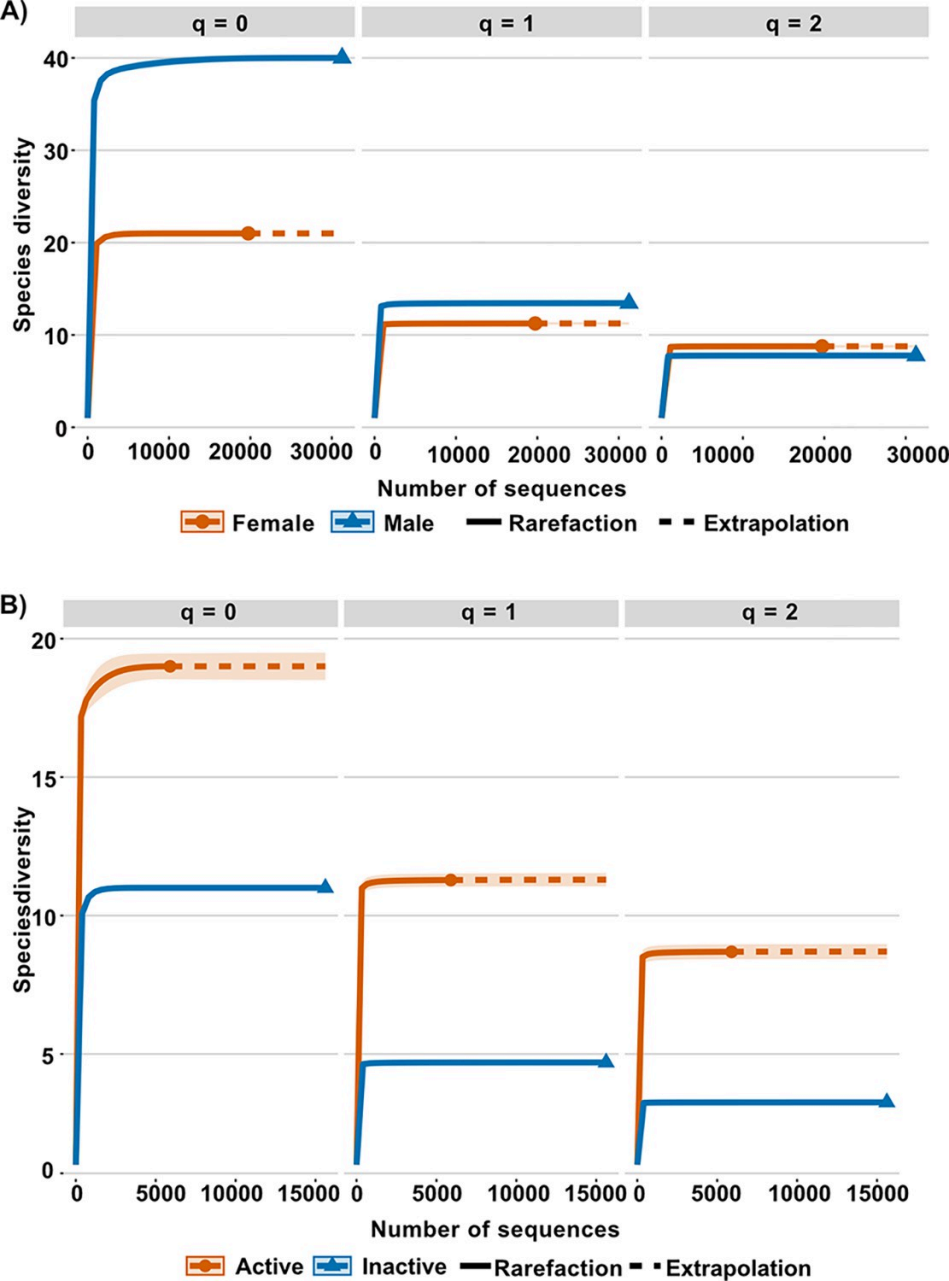
Hill numbers (q0 = richness, q1 = effective number of common genera, q2 = effective number of dominant genera, per sex and by sexual condition in males). (A) Rarefaction curves based on the number of sequences per sex. (B) Rarefaction curves based on the number of sequences per sexual condition in males (active and inactive).

The richness of males (q0 = 40) was twice as high as that of females (q0 = 21), while the equitability (q1 female = 11.24, q1 male = 13.44) and dominance (q2 female = 8.76, q2 male = 7.76) of species was similar in both sexes (Fig 4A).

The richness of sexually active males (q0 = 19) was twice that of sexually inactive males (q0 = 11), while the equitability (q1 active = 11.28, q1 inactive = 4.69) and dominance (q2 active = 8.69, q2 inactive = 3.25) of sexually active males was three times that of sexually inactive males (Fig 4B).

### Beta diversity analysis

**Fig 5:**
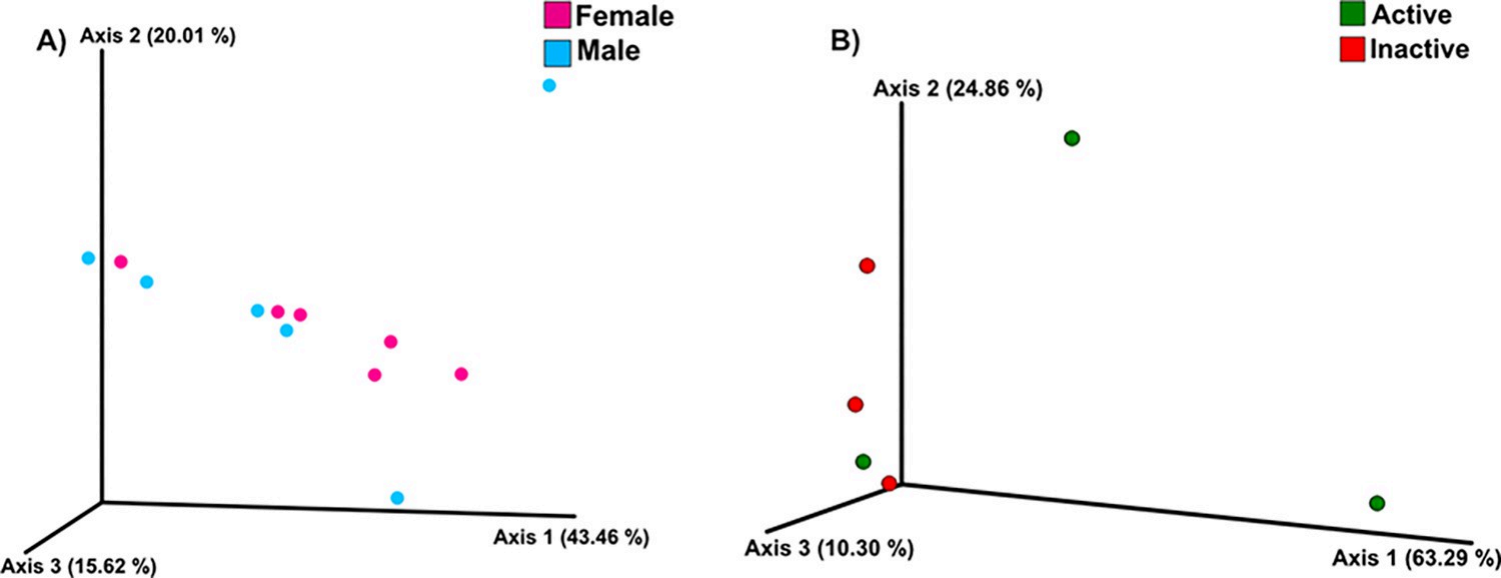
Principal Coordinates Analysis (PCoA) of the beta diversity per sex and between sexually active and inactive males. (A) PCoA of the beta diversity present in the fecal microbiota from *M*. *velifer* males and females, using weighted unifrac distance. (B) PCoA of the beta diversity present in the fecal microbiota from *M*. *velifer* sexually active and inactive males, using weighted unifrac distance.

Principal coordinate analysis (PCoA) of phylogenetic beta diversity using weighted Unifrac distance as a quantitative measure of dissimilarity, revealed no differences between the fecal microbiota of males and females of the *M*. *velifer* (Fig 5A). Additionally, PCoA of phylogenetic beta diversity indicated no differences in the fecal microbiota of sexually active versus inactive males (Fig 5B).

**Fig 6:**
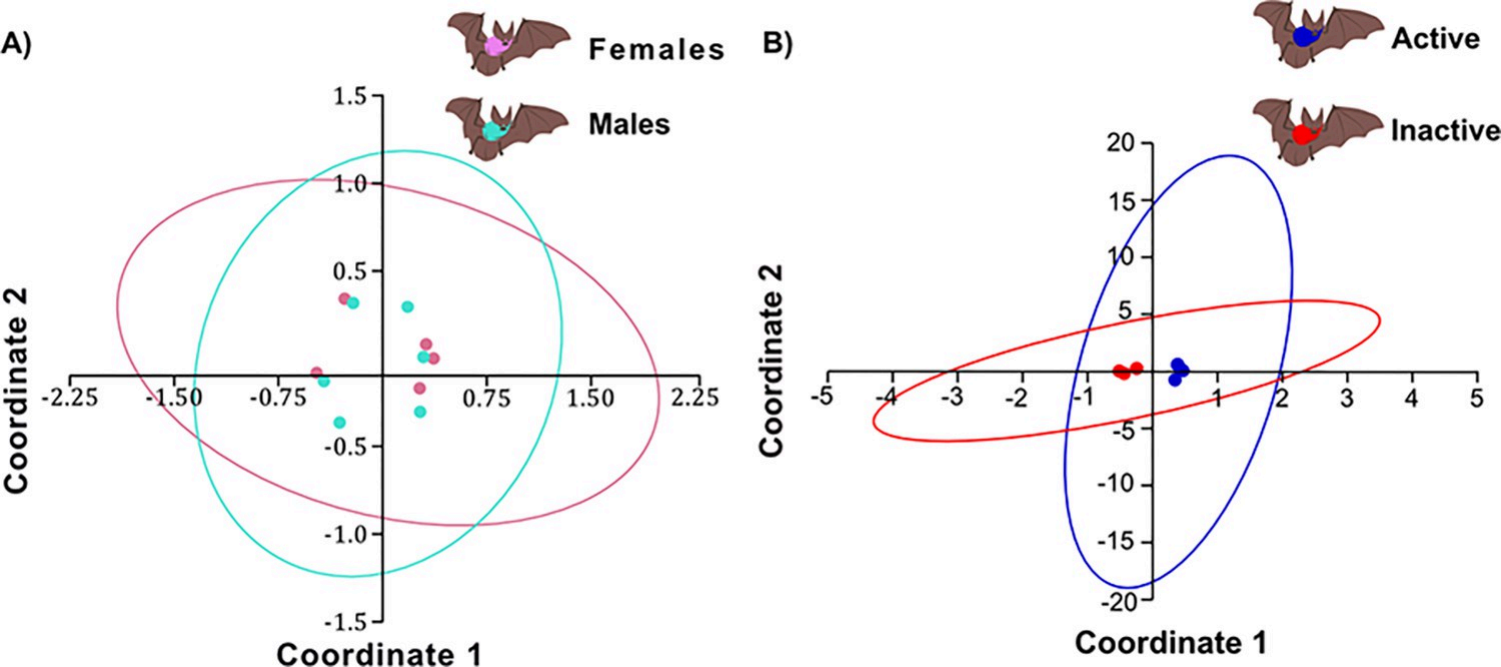
ANOSIM between sex and sexually active and inactive males. (A) Similarity analysis between males (blue) and females (pink) (ANOSIM). (B) Similarity analysis between sexually active (blue) and inactive (red) males (ANOSIM).

The analysis of similarity between groups (ANOSIM) indicated no significant differences in beta diversity between male and female groups (r = 0.08267, p = 0.7543; Fig 6A). Similarly, ANOSIM analysis revealed no significant differences in beta diversity between sexually active and inactive males (r = 0.2778, p = 0.2052; Fig 6B).

## Discussion

There are several studies on the fecal microbiota of wild insectivorous bats that may be summarized as follows: (i) the most studied bat genera have been *Myotis*, *Rhinolophus* and *Hipposideros*, (ii) the bacterial phyla that dominate are Pseudomonadota and Bacillota, (iii) the main bacterial genera reported have been *Pleisomonas*, *Enterococcus*, *Lactococcus* and *Lactobacillus*, and iv) most of the feces collected for these studies belong to bats distributed in China [21, 24, 26, 40, 46, 47]. However, to our knowledge, this is the first investigation to characterize the fecal microbiota of *M*. *velifer*, an insectivorous bat distributed mainly in Mexico. The mouse-eared bat establishes symbiotic relationships with communities of microorganisms (such as bacteria, archaea and fungi) [3] that make up its gut microbiota.

Here we report that the fecal microbiota of the bat *M*. *velifer* is dominated by the phyla Pseudomonadota, Fusobacteriota, Bacillota and Synergistota. This finding is consistent with previous studies of the intestinal and fecal microbiota found for other bats. For example, in the gut microbiota of phytophagous (nectarivorous and frugivorous) and insectivorous bats from southern China, the microbiota was dominated by Pseudomonadota, Bacillota and Bacteroidota [24, 26]. In lesser horseshoe bats Pseudomonadota dominates, followed by Bacillota [47]. The fecal bacterial microbiota of the insectivorous bat *Mops condylurus* had Bacillota and Pseudomonadota [40] and in two insectivorous bats (*Rhinolophus sinicus* and *Myotis altarium*) in three different sampling sources (small intestine, large intestine and feces) the most dominant phylum was Pseudomonadota, while Fusobacteriota was the least dominant [21]. Unlike the results obtained from the research of Wu and collaborators [21], in this study we found that the phylum Fusobacteriota was the second dominant phylum with 23% of the fecal microbiota of *M*. *velifer*.

This study is the first report of the Synergistota phylum in bat microbiota. This phylum, which comprised 5% of the total fecal microbiota of *M*. *velifer*, has been described only in the intestinal tract of pigs [69], termites [70] and in the human mouth [71]. This group of bacteria is characterized by anaerobic degraders of amino acids [72–74].

Regarding the composition at the family level, 24 families were identified, with Fusobacteriaceae, Rhodospirillaceae and Enterobacteriaceae being the most abundant. Fusobacteriaceae and Enterobacteriaceae are characterized by being potentially opportunistic pathogens and are reported in different proportions in the oral, gastrointestinal [21, 75] and fecal microbiota of bats [24]. On the other hand, Rhodospirillaceae has not been reported in bats, and this group of bacteria has a wide variety of habitats, ranging from plant tissues, soil contaminated with oil to aquatic environments such as oceans and stagnant water [76].

Our results highlight the dominance of the genus *Cetobacterium* in the microbiota of *M*. *velifer*. It has also been reported as a dominant genus in the fecal microbiota of freshwater fish, where they function as producers of vitamin B12, which is distributed throughout the intestinal tract [77, 78]. Therefore, it can be speculated that they perform the same function in the intestinal tract of *M*. *velifer*, since this vitamin is not synthesized by animals or plants, but by some bacteria and archaea [79]. It is worth mentioning that this genus has been isolated from feces of marine mammals [80] and humans [81]. Future studies should focus on the role of *Cetobacterium* in bats and the implications it has for their ecology.

The second dominant genus from the *M*. *velifer* fecal microbiota is *Haematospirillum*, isolated for the first time in human blood [82] and later found in the blood of an insectivorous bird [83]. It is described as a possible emerging infectious pathogen for humans [82, 84, 85]. Our finding constitutes the first molecular evidence of this species in the fecal microbiota of bats. *Haematospirillum* may produce non-volatile organic acids and may be involved in degrading organic substrates containing sulfur [82, 86, 87].

Regarding reproductively active and inactive males, differences in composition and relative abundances were observed as described before in the fecal microbiota of different reproductive stages of the nectivorous bat *Leptonycteris yerbabuenae* [8]. Other bacterial genera in the fecal microbiota of active and inactive individuals were *Caccocola* which was identified through metagenomic analyzes in chicken feces [88], *Entomobacter*, isolated from the intestine of the Madagascar hissing cockroach (*Gromphadorhina portentosa*) [89], *Enterocloster* bacteria described as opportunistic pathogens and commonly found in the intestine of humans [90] and *Saezia*, belonging to the order Burkholderiales [91]. These bacterial genera are newly described here for bats in the Americas.

From males, we observed seven phyla, that included 21 of the 24 total families and 26 of the 31 total genera, that is, 84% of the richness described for the fecal microbiota of the bat *M*. *velifer*. On the other hand, in females, four of seven phyla, 12 of 24 families and 14 of 31 genera were identified, which is equivalent to 45% of the total richness characterized. It should be noted that Pseudomonadota was the most abundant phylum consistent with the results of Banskar [45]. Fusobacteriaceae and *Cetobacterium* were the dominant family and genus for *Myotis velifer*, however, between sexes, the relative abundance values change.

Alpha diversity analyses revealed significant differences between sexes and among male reproductive stages (active and inactive). Previous analyses of the microbiota of phyllostomid bats, comparing sexes and reproductive conditions within sexes, have generally shown that both reproductive males and females harbor the most diverse bacterial communities [8, 48]. However, in our study, reproductively active males exhibited a more diverse fecal microbiota compared to both females and inactive males. According to Riopelle [48], this finding underscores the urgent need for further studies on bacteria not shared by male and female bats to clarify sex differences.

In contrast to the alpha diversity analyses, the beta diversity analyses (UNIFRAC, PCoA and ANOSIM) indicated no significant differences between the sexes or between sexually active and inactive males. This lack of beta diversity differences may suggest that, although variation exists in the abundance and distribution of bacterial genera across groups, they share a fecal microbiota with a similar composition. This could indicate the presence of a stable bacterial community that does not differ structurally at the genus level, likely due to shared factors such as diet, environment, and lifestyle [92].

## Conclusions

The detailed study on the fecal microbiota of the *Myotis velifer* bat using massive amplicon sequencing techniques revealed a diverse and distinctive composition compared to the microbiota from other previously studied bats. The dominance of Pseudomonadota, Fusobacteriota, Bacillota and Synergistota, together with the identification of 33 families and the prevalence of genera such as *Cetobacterium* and *Haematospirillum* revealed the uniqueness of the microbiota of this insectivore which has an extensive geographical distribution.

Although some similarities were observed with previous studies in bats, such as the prevalence of Pseudomonadota, this study highlights notable differences, such as the significant presence of Fusobacteriota. On the other hand, the identification of differences in the composition of the microbiota between males and females of *M*. *velifer* is notable, despite the absence of significant differences in beta diversity analyses.

## Supporting information

S1 Fig Bacterial families from the fecal microbiota of *Myotis velifer* per sex. Percentage of relative abundance of the families from domain Bacteria in the fecal microbiota of *M*. *velifer*, with the most abundant at the top and the least abundant at the bottom.
(TIF)

S2 Fig Bacterial genera per individual found in the fecal microbiota of *Myotis velifer*. Percentage of relative abundance of the genera from domain Bacteria in the fecal microbiota of *M*. *velifer*, with the most abundant at the top and the least abundant at the bottom.
(TIF)

S3 Fig Sexually active and inactive males (per individual). Percentage of relative abundance of the genera from domain Bacteria in active and inactive males, with the most abundant at the top and the least abundant at the bottom.
(TIF)
