# Supplementary figures and images for: The fecal microbiota of the mouse-eared bat (Myotis velifer) with new records of microbial taxa for bats
Source: PLoS One. 2024 Dec 5;19(12):e0314847. doi: 10.1371/journal.pone.0314847 (PMC11620696; doi:10.1371/journal.pone.0314847)

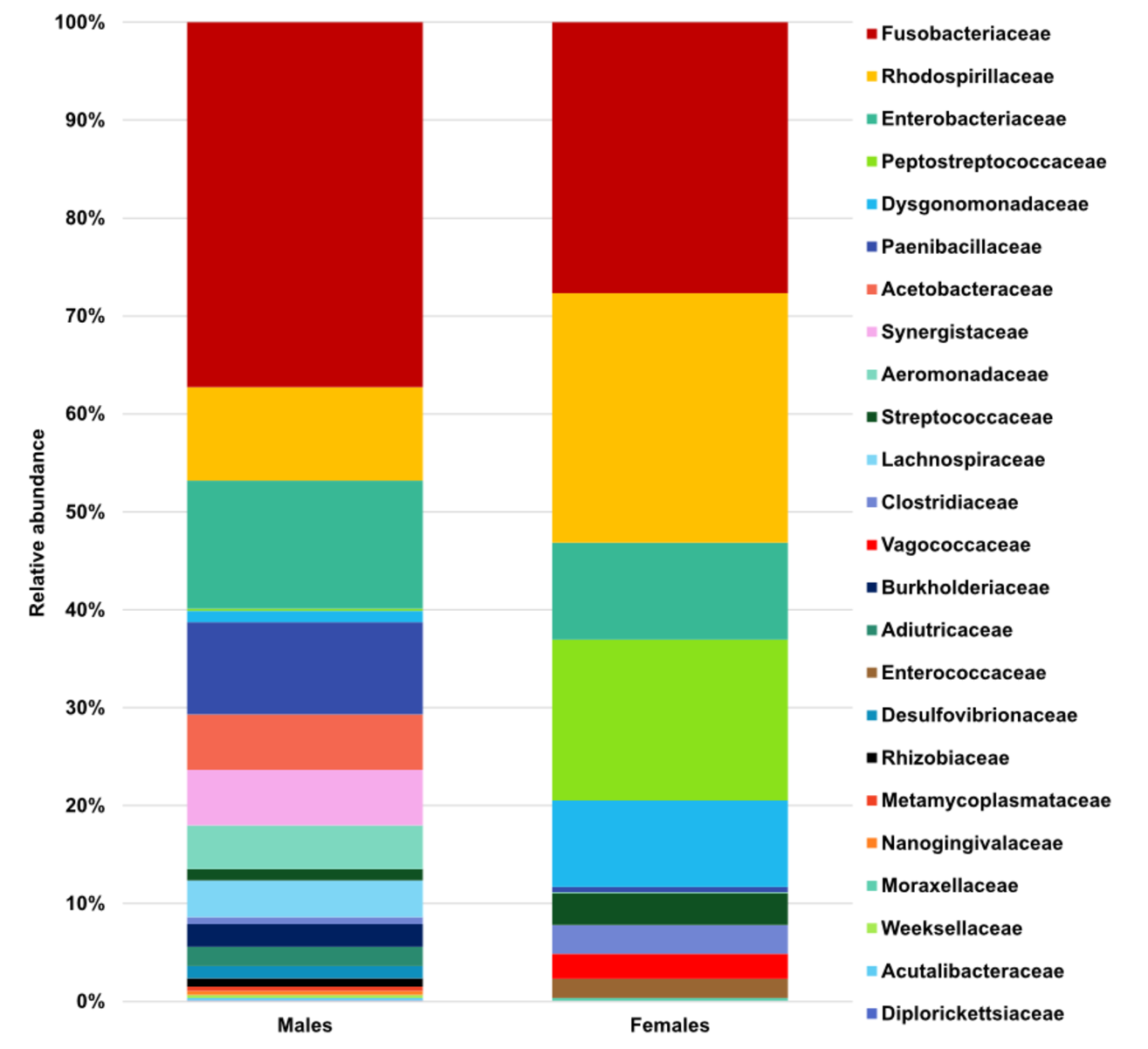

Supplement: S1 Fig — Percentage of relative abundance of the families from domain Bacteria in the fecal microbiota of M. velifer, with the most abundant at the top and the least abundant at the bottom. (TIF) [file pone.0314847.s001.tif]

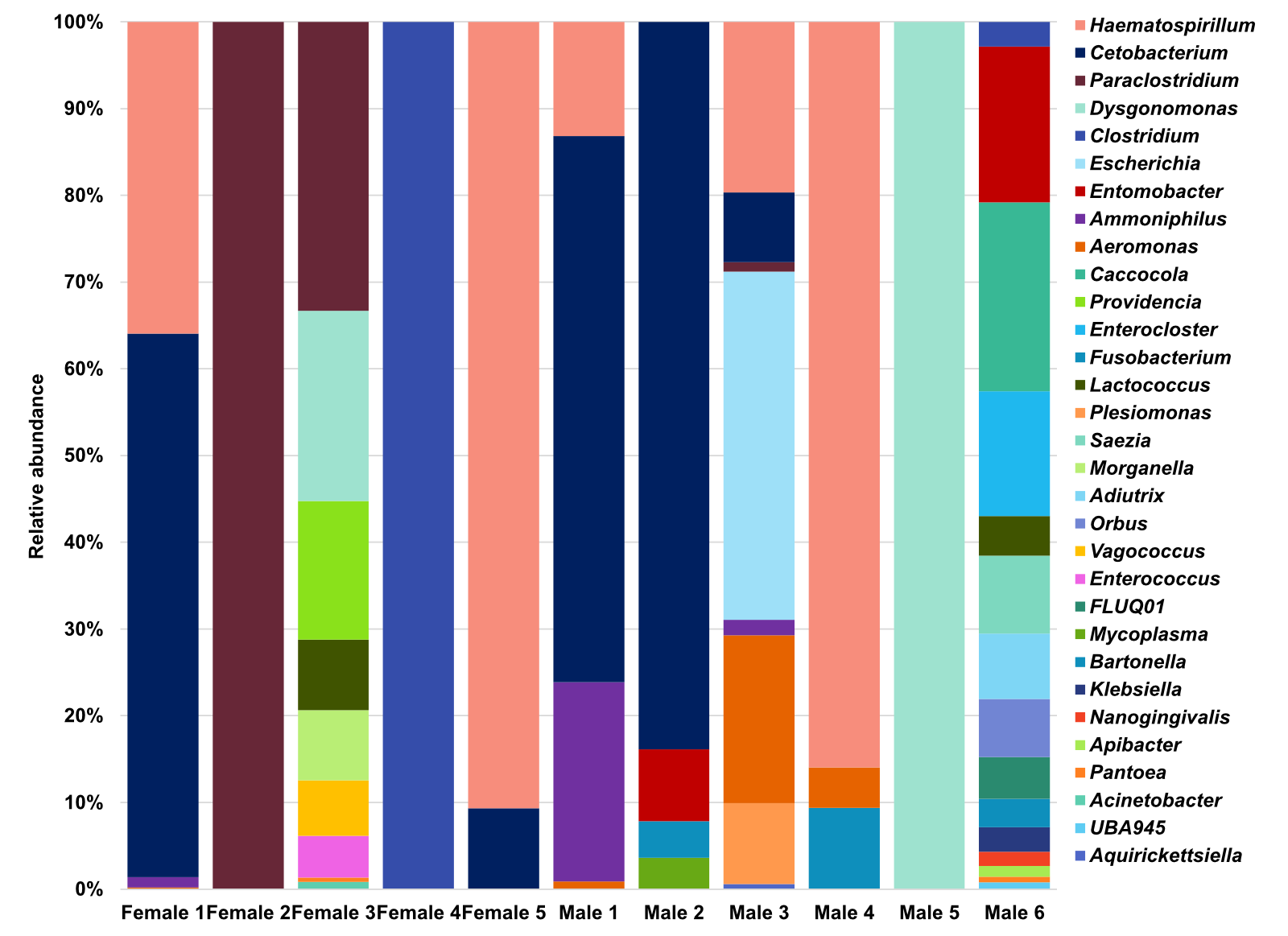

Supplement: S2 Fig — Percentage of relative abundance of the genera from domain Bacteria in the fecal microbiota of M. velifer, with the most abundant at the top and the least abundant at the bottom. (TIF) [file pone.0314847.s002.tif]

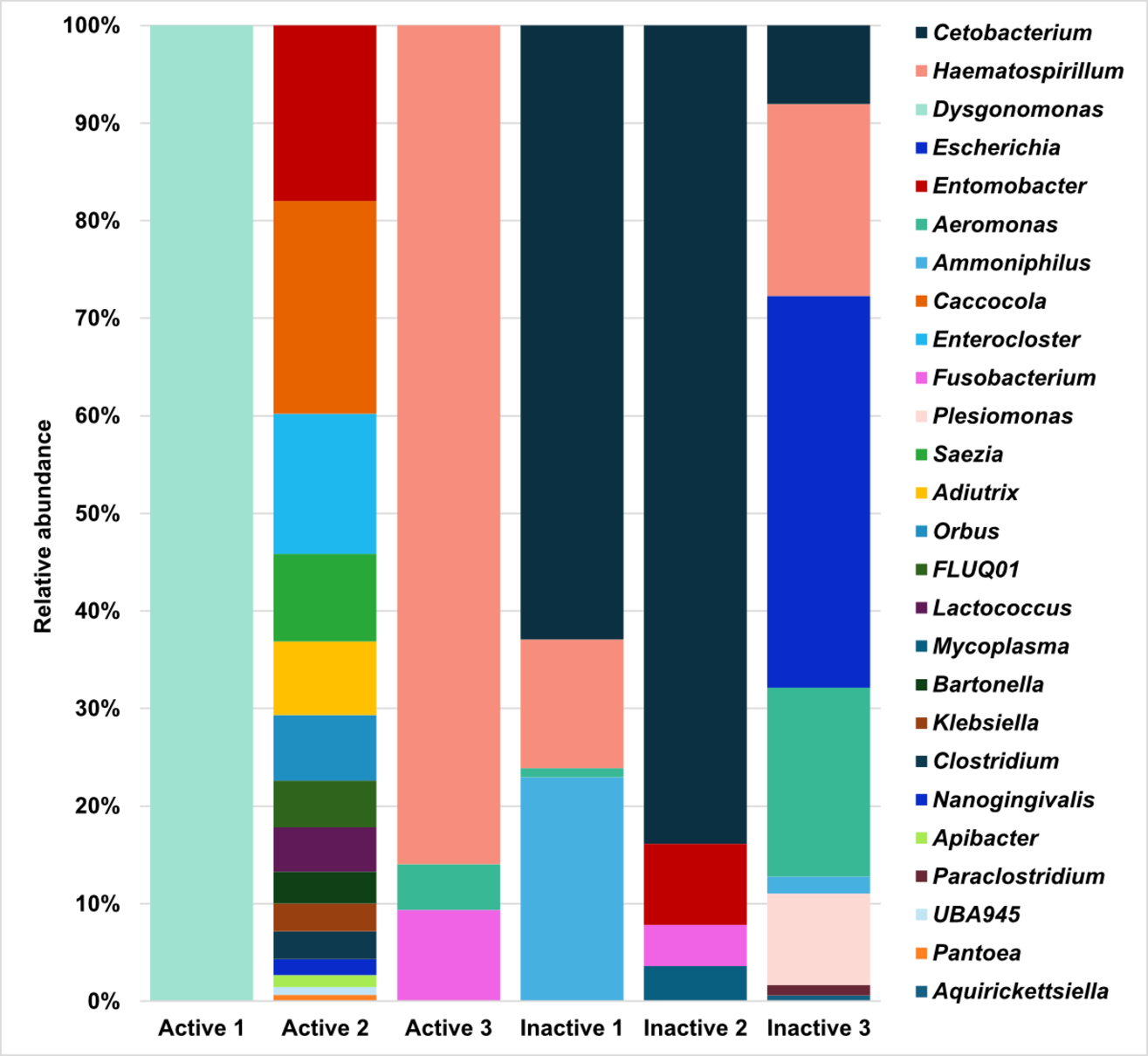

Supplement: S3 Fig — Percentage of relative abundance of the genera from domain Bacteria in active and inactive males, with the most abundant at the top and the least abundant at the bottom. (TIF) [file pone.0314847.s003.tif]
